# Safety Profile of Intranasal Corticosteroids in Allergic Rhinitis: A Comprehensive Review

**DOI:** 10.3390/biomedicines14071536

**Published:** 2026-07-09

**Authors:** Mirko Maglica, Franko Batinović, Marin Gudelj, Braco Bošković, Ivan Mizdrak, Stjepan Radić, Marta Knežević, Ivan Paladin

**Affiliations:** Department of Otorhinolaryngology, University Hospital of Split, 21000 Split, Croatia; fbatinovic1@gmail.com (F.B.); marin.gudelj8@gmail.com (M.G.); bboskovic01@gmail.com (B.B.); imizdrak@yahoo.com (I.M.); strradic@gmail.com (S.R.); marta.knezevic7@gmail.com (M.K.); ivan.paladin@gmail.com (I.P.)

**Keywords:** intranasal corticosteroids, epistaxis, systemic bioavailability, pediatric safety, HPA axis

## Abstract

Intranasal corticosteroids (INCS) remain the cornerstone of pharmacologic treatment for allergic rhinitis (AR) because of their well-established anti-inflammatory efficacy and generally favorable benefit–risk profile. Nevertheless, concerns regarding local and systemic corticosteroid-related adverse events (AEs) continue to influence patient adherence, prescribing practices, and long-term treatment acceptance. In routine clinical practice, safety perception and corticosteroid-related concerns frequently influence adherence and formulation selection to a greater extent than differences in clinical efficacy, particularly in pediatric populations and in patients requiring prolonged continuous therapy. Differences in pharmacokinetic and pharmacodynamic properties, including systemic bioavailability, glucocorticoid receptor affinity, lipophilicity, protein binding, and extent of first-pass metabolism, are considered important safety profile determinants of currently available INCS formulations. Available evidence indicates that local AEs, particularly epistaxis, nasal irritation, dryness, and sensory discomfort, represent the most frequently reported treatment-related AEs across INCS formulations, although these events are generally mild, self-limiting, and infrequently treatment-limiting. Clinically significant structural nasal complications, including septal perforation or progressive mucosal injury, appear uncommon in currently available studies. Systemic AEs, including hypothalamic–pituitary–adrenal (HPA) axis suppression, ocular toxicity, growth impairment, or clinically meaningful effects on bone metabolism, have not been consistently demonstrated with currently used low-systemic-exposure formulations administered at recommended therapeutic doses. Although systemic glucocorticoid exposure has been associated with alterations in lipid metabolism, adipose tissue function, and metabolic homeostasis, currently available intranasal corticosteroids demonstrate minimal systemic exposure, making clinically relevant metabolic effects unlikely under recommended therapeutic conditions. Formulations such as mometasone furoate, fluticasone propionate, fluticasone furoate, and ciclesonide exhibit pharmacokinetic characteristics associated with minimal systemic exposure because of extensive first-pass metabolism and low oral bioavailability. Although substantial pharmacokinetic differences exist between currently available INCS formulations, direct comparative evidence demonstrating clinically meaningful superiority in systemic safety outcomes remains limited. Current evidence suggests that formulation-dependent differences are clinically more relevant with respect to local tolerability, sensory characteristics, patient preference, and long-term adherence than major systemic safety outcomes. Pediatric evidence is generally reassuring, although historical concerns regarding growth suppression associated with earlier corticosteroid formulations continue to influence clinical practice. Currently available evidence supports the use of modern INCS as effective and generally well-tolerated therapeutic options across adult and pediatric populations.

## 1. Introduction

Allergic rhinitis (AR) is one of the most prevalent chronic inflammatory airway diseases and is associated with substantial impairment in quality of life, sleep, cognitive performance, and work productivity. Intranasal corticosteroids (INCS) remain the cornerstone of pharmacologic management because of their superior efficacy in controlling nasal inflammation and improving overall symptom burden [1]. Clinical efficacy is typically evaluated using validated patient-reported outcome measures such as the Total Nasal Symptom Score (TNSS), Total Ocular Symptom Score (TOSS), and Rhinoconjunctivitis Quality of Life Questionnaire (RQLQ), which are widely accepted despite their subjective nature [2,3,4,5,6].

Despite their well-established efficacy, long-term adherence to INCS therapy remains suboptimal in routine clinical practice. Concerns regarding corticosteroid-associated adverse events (AEs), sensory discomfort, local irritation, and perceived systemic toxicity frequently contribute to treatment discontinuation or poor adherence, particularly among pediatric patients and caregivers [7,8,9,10,11,12,13]. Consequently, safety perception may influence treatment persistence and real-world effectiveness as strongly as pharmacologic efficacy itself [7,8]. A recent meta-epidemiological analysis of completed AR randomized controlled trials (RCTs) further emphasized the importance of systematic AE evaluation across pharmacologic treatment classes and identified INCS as the intranasal therapy group with the most favorable overall safety profile [14].

Historically, safety concerns were more prominent with earlier corticosteroid formulations characterized by greater systemic exposure, whereas currently available INCS have been developed to optimize topical anti-inflammatory activity while minimizing systemic absorption. Pharmacokinetic and pharmacodynamic properties, including glucocorticoid receptor affinity, lipophilicity, protein binding, oral bioavailability, and extent of first-pass metabolism, are therefore considered important determinants of formulation-specific safety profiles [15,16,17,18,19,20]. Accordingly, understanding both shared class effects and formulation-specific safety characteristics is essential for individualized treatment selection, particularly in pediatric populations and in patients requiring long-term therapy. Differences in sensory tolerability, spray characteristics, and patient preference may additionally influence long-term adherence and real-world treatment effectiveness [7,9,11,12]. Beyond their anti-inflammatory effects, corticosteroids may influence lipid metabolism and metabolic homeostasis when systemic exposure occurs; therefore, understanding the extent to which modern intranasal formulations minimize systemic absorption has relevance not only for traditional corticosteroid-related adverse events but also for potential metabolic safety considerations [21,22].

This review summarizes current evidence regarding local and systemic safety of currently available INCS formulations, with particular emphasis on pharmacologic determinants of safety, formulation-dependent tolerability, pediatric considerations, and the clinical relevance of pharmacokinetic differences between currently available agents.

## 2. Methods and Literature Search Strategy

This narrative review was conducted to summarize current evidence regarding the safety profile of intranasal corticosteroids (INCS) used in the management of allergic rhinitis. A literature search was performed using PubMed/MEDLINE, Scopus, and Google Scholar to identify relevant publications addressing the pharmacology, pharmacokinetics, efficacy, safety, and adverse events associated with currently available INCS formulations. Search terms included combinations of “intranasal corticosteroids”, “allergic rhinitis”, “safety”, “adverse events”, “systemic bioavailability”, “pharmacokinetics”, “epistaxis”, “hypothalamic-pituitary-adrenal axis”, “growth”, “ocular effects”, and individual corticosteroid names (e.g., mometasone furoate, fluticasone propionate, fluticasone furoate, budesonide, triamcinolone acetonide, beclomethasone dipropionate, and ciclesonide).

Priority was given to randomized controlled trials, meta-analyses, systematic reviews, pharmacokinetic studies, and clinical practice guidelines published in English. Additional relevant studies were identified through manual screening of reference lists from key publications. Studies were selected based on their relevance to predefined topics including pharmacologic determinants of safety, local adverse events, systemic adverse events, pediatric safety considerations, formulation characteristics, and clinical implications of pharmacokinetic differences. As this manuscript represents a narrative review, no formal systematic-review protocol or quantitative meta-analysis was performed. Ultimately, 113 publications were included in the qualitative narrative synthesis, comprising randomized controlled trials, pharmacokinetic studies, systematic reviews, meta-analyses, clinical practice guidelines, and selected narrative reviews relevant to the predefined topics.

## 3. Pharmacologic Determinants of Safety

### 3.1. Systemic Bioavailability and First-Pass Metabolism

The systemic safety profile of INCS is influenced largely by pharmacokinetic characteristics including systemic bioavailability, glucocorticoid receptor affinity, lipophilicity, protein binding, and extent of first-pass hepatic metabolism [8,15,19,23]. These properties affect the balance between topical anti-inflammatory efficacy and systemic corticosteroid exposure.

Mometasone furoate (MF) demonstrates particularly low systemic bioavailability because of extensive first-pass metabolism and minimal gastrointestinal absorption of the swallowed fraction [24,25,26,27,28]. Similarly, fluticasone propionate (FP) undergoes extensive first-pass hepatic metabolism and exhibits negligible oral bioavailability, resulting in minimal systemic exposure following intranasal administration [29,30,31]. Fluticasone furoate (FF) additionally demonstrates high glucocorticoid receptor binding affinity, which may contribute to sustained local anti-inflammatory activity at relatively low doses [32,33]. Ciclesonide differs mechanistically from other INCS because it is administered as an inactive prodrug that undergoes enzymatic conversion within the nasal epithelium to its active metabolite desisobutyryl-ciclesonide (des-CIC) [34,35,36,37]. Both ciclesonide and des-CIC exhibit minimal gastrointestinal absorption, high plasma protein binding, and extensive cytochrome P450 3A4 (CYP3A4) mediated first-pass metabolism, resulting in low systemic bioavailability and limited systemic exposure [15,34,38,39].

Compared with very low-bioavailability formulations such as MF or FP nasal sprays, older agents, including budesonide and triamcinolone acetonide, demonstrate less favorable pharmacokinetic profiles concerning systemic exposure, although clinically meaningful systemic toxicity remains uncommon at recommended intranasal doses [40,41]. Importantly, marked pharmacokinetic differences between formulations do not necessarily translate into clinically meaningful differences in systemic safety outcomes under standard therapeutic conditions [20,42]. Available comparative studies generally demonstrate low rates of clinically significant systemic AEs across approved INCS formulations when administered at recommended doses.

Collectively, currently available pharmacokinetic and pharmacodynamic data suggest that modern low-systemic-exposure INCS substantially limit systemic corticosteroid exposure while maintaining effective local anti-inflammatory activity.

### 3.2. Receptor Affinity and Lipophilicity

Glucocorticoid receptor binding affinity and lipophilicity may further influence local anti-inflammatory potency and tissue retention. FF and MF exhibit particularly high receptor affinity, whereas des-CIC demonstrates strong receptor binding following enzymatic activation within the nasal mucosa [32,33,34,43,44]. These characteristics may contribute to prolonged mucosal retention and sustained topical anti-inflammatory activity while maintaining minimal systemic exposure [32,33,45]. Although these pharmacodynamic properties may theoretically improve topical efficacy and prolong local residence time, currently available comparative studies generally demonstrate broadly similar clinical efficacy between therapeutically equivalent INCS formulations [9,20,46]. Consequently, differences in receptor affinity do not necessarily translate into clinically meaningful superiority in safety or efficacy outcomes.

### 3.3. Formulation Characteristics and Nasal Deposition

Formulation characteristics may additionally influence local tolerability and patient preference. Hypotonic aqueous formulations such as ciclesonide aqueous nasal spray may enhance mucosal diffusion and reduce nasal runoff [47,48], whereas isotonic formulations such as FP nasal spray may be associated with greater postnasal drip and runoff [10,47]. Delivery systems including aqueous sprays, aerosols, and hydrofluoroalkane-propelled formulations may further affect nasal deposition patterns, sensory characteristics, and local AE profiles [49,50]. Real-world tolerability may also be influenced by factors unrelated to corticosteroid potency itself, including excipient-related mucosal irritation associated with preservatives such as benzalkonium chloride [51]. Additional factors, including spray technique, administration angle, spray volume, excipient composition, odor perception, taste disturbance, and postnasal drainage, may further influence patient perception and treatment adherence [7,9,52]. Incorrect spray technique directed toward the nasal septum may contribute to local irritation and epistaxis independently of formulation-specific pharmacologic properties [52]. Such formulation-dependent differences may contribute meaningfully to treatment adherence during long-term therapy despite generally comparable anti-inflammatory efficacy. Several studies additionally demonstrated rapid onset of symptom improvement with ciclesonide formulations, which may further influence patient satisfaction and adherence during routine clinical use [53,54].

### 3.4. Corticosteroids, Lipid Metabolism, and Potential Metabolic Consequences of Systemic Exposure

Beyond their established anti-inflammatory effects, glucocorticoids play an important role in the regulation of energy homeostasis and lipid metabolism. Through activation of glucocorticoid receptors in adipose tissue, liver, and skeletal muscle, glucocorticoids influence adipocyte differentiation, lipogenesis, lipolysis, fatty acid mobilization, and lipid storage [21,22,55]. Prolonged systemic exposure to corticosteroids has been associated with metabolic alterations including increased visceral adiposity, dyslipidemia, insulin resistance, and features of metabolic syndrome, highlighting the importance of minimizing unnecessary systemic corticosteroid exposure whenever possible [22,55].

The mechanisms underlying these effects are complex and involve both genomic and non-genomic pathways. Glucocorticoids regulate the expression of genes involved in lipid turnover and adipocyte function, while also interacting with metabolic signaling pathways that influence glucose utilization and fatty acid metabolism [22]. In adipose tissue, corticosteroids may promote regional fat redistribution and alter the balance between lipid storage and mobilization, effects that become clinically relevant primarily during long-term systemic therapy [55].

From the perspective of intranasal corticosteroid safety, these observations reinforce the importance of pharmacokinetic characteristics that limit systemic bioavailability. Modern INCS formulations such as mometasone furoate, fluticasone propionate, fluticasone furoate, and ciclesonide exhibit extensive first-pass metabolism and minimal systemic exposure following intranasal administration [15,16,19,46]. Consequently, although systemic glucocorticoid therapy may influence lipid metabolism and metabolic homeostasis, currently available evidence does not demonstrate clinically meaningful effects of recommended-dose INCS therapy on lipid metabolism or related metabolic outcomes. The negligible systemic exposure achieved with contemporary low-bioavailability formulations is therefore considered an additional factor supporting their favorable long-term safety profile.

## 4. Local AEs and Formulation-Dependent Tolerability

### 4.1. Epistaxis

Epistaxis was among the most frequently reported local AEs in studies of beclomethasone dipropionate (BDP) [56,57], triamcinolone acetonide (TAA) [58], FP nasal spray [11,12,59], MF nasal spray [60,61], FF nasal spray [62,63,64,65,66,67,68], and ciclesonide formulations [69,70,71,72]. In these studies, events were generally mild, self-limiting, and infrequently associated with treatment discontinuation [68,72,73]. Reported incidence rates varied considerably between studies, likely reflecting differences in study duration, patient populations, definitions of epistaxis, and AE reporting methodology. Many studies additionally relied on spontaneous AE reporting without standardized grading systems, limiting direct comparison between formulations and studies. Most short-term randomized studies reported epistaxis rates below 10% and generally comparable to placebo [5,8,9,25,57,66,67] whereas some long-term FF nasal spray studies reported rates approaching 15–20%, although severe bleeding events remained uncommon [63,64,68]. Nevertheless, severe epistaxis and clinically significant bleeding events appear uncommon across currently available formulations [40,69,70,71]. Overall, available evidence suggests that epistaxis associated with INCS therapy is generally mild, manageable, and infrequently associated with clinically significant structural injury. Although meta-analyses of INCS trials have demonstrated an increased relative risk of epistaxis compared with placebo, reported events are generally mild, self-limiting, and rarely clinically significant [14,73].

### 4.2. Nasal Irritation, Dryness, and Sensory Effects

Local irritation-related symptoms, including burning, stinging, dryness, sneezing, throat discomfort, and nasal discomfort, were reported in studies of BDP [57,71], budesonide [74], TAA [41,49], FP nasal spray [11,12,47,48], MF nasal spray [60,73], and ciclesonide [48]. Across these studies, symptoms were generally transient, mild to moderate in severity, and rarely clinically significant. Sensory tolerability appears to be influenced primarily by formulation characteristics rather than corticosteroid potency. Aerosol delivery systems, hypotonic formulations, and differences in spray volume or excipients may substantially affect patient perception and treatment preference [12,47,48]. FP nasal spray formulations were associated with greater odor perception and postnasal runoff in some comparative studies [11,12,47,75], whereas hypotonic ciclesonide formulations may reduce nasal dripping and throat deposition [47,48]. However, some crossover studies reported higher rates of nasal irritation with ciclesonide than with FP nasal spray [12]. Differences in sensory attributes may be clinically relevant because patient preference can influence adherence during long-term treatment [11,12].

### 4.3. Structural Nasal Complications

Clinically significant structural nasal complications associated with INCS therapy appear uncommon. Pediatric BDP studies reported no cases of nasal septal perforation, with only rare nasal septum disorders observed [57]. MF nasal spray studies demonstrated preservation of mucosal integrity and, in some studies, improvement in the proportion of intact ciliated epithelium during treatment [76,77,78]. Long-term FF nasal spray studies identified occasional mucosal bleeding, crusting, and ulceration compared with placebo, although progressive structural damage or clinically significant mucosal atrophy was not observed [62,63,79]. Similarly, ciclesonide studies reported nasal erosions in fewer than 1.5% of patients and no cases of septal perforation [48,80]. Available studies generally identified mild mucosal irritation or superficial bleeding rather than progressive structural tissue injury.

## 5. Systemic Safety

### 5.1. Hypothalamic–Pituitary–Adrenal Axis Function

Suppression of the hypothalamic–pituitary–adrenal (HPA) axis has historically represented one of the principal theoretical safety concerns associated with corticosteroid therapy. However, available pharmacodynamic studies evaluating currently used INCS generally have not demonstrated clinically meaningful suppression of HPA axis function at recommended therapeutic doses [29,31,45,48,81,82,83,84,85,86,87,88]. Studies of BDP [42,81], TAA [41], FP nasal spray [29,30,31], MF nasal spray [82,83], FF nasal spray [45,68,84,85,86,87], and ciclesonide [88] reported serum cortisol concentrations, urinary free cortisol measurements, and dynamic assessments of adrenal function that were generally comparable to placebo in both adult and pediatric populations. Earlier studies involving older corticosteroids such as BDP and budesonide raised concerns regarding systemic corticosteroid exposure, particularly during prolonged pediatric use [89,90,91,92,93,94]. Nevertheless, clinically significant HPA axis suppression appears uncommon with currently recommended intranasal dosing regimens [81,84,87]. Importantly, interpretation of HPA axis findings is complicated by differences in cortisol assessment methodology, timing of sampling, study duration, and sensitivity of adrenal function testing between studies [20,95]. Many RCTs were additionally underpowered to detect rare systemic effects or subtle cumulative endocrine changes associated with prolonged exposure.

### 5.2. Ocular Safety

Potential corticosteroid-associated ocular complications, including glaucoma, cataracts, and elevated intraocular pressure, remain important theoretical concerns during long-term INCS therapy. However, available studies of budesonide, MF nasal spray, FF nasal spray, and ciclesonide did not demonstrate a consistent association with clinically meaningful ophthalmologic toxicity [68,70,71,79,82,85]. Across these studies, intraocular pressure measurements generally remained stable and comparable to placebo, while clinically significant cataract formation or progressive lens abnormalities were not observed [73,74,82,83,85].

### 5.3. Bone Metabolism and General Systemic Toxicity

Studies evaluating FP nasal spray [29,31,59], TAA [41,49], FF nasal spray [62,63,96], and ciclesonide [69,70,71] did not identify clinically meaningful systemic toxicity involving laboratory parameters, cardiovascular findings, or generalized corticosteroid-related AEs. Potential effects on bone metabolism have been discussed primarily in the context of inhaled corticosteroid therapy [95,97,98]. Although reductions in bone mineral density and alterations in biochemical markers of bone turnover have been reported with inhaled corticosteroids [95], comparable findings have not been consistently demonstrated with intranasal therapy [97,98]. Overall, currently available studies suggest that INCS are associated with minimal clinically significant systemic toxicity when administered intranasally at therapeutic doses.

### 5.4. Drug Interactions and Rare Systemic Complications

Although clinically significant systemic AEs are uncommon with INCS, clinicians should remain aware of the potential for pharmacokinetic interactions involving strong CYP3A4 inhibitors, particularly in patients receiving multiple corticosteroid-containing therapies. However, most clinically significant interaction data originate from inhaled rather than intranasal corticosteroid use [42]. Nevertheless, cumulative corticosteroid exposure may become clinically relevant in patients receiving concomitant inhaled, topical, oral, or ophthalmic corticosteroid therapies, particularly during prolonged treatment or in pediatric populations [20,89].

### 5.5. Special Populations and Clinical Considerations

Although pediatric safety has been extensively evaluated, evidence regarding the use of INCS in other special populations remains comparatively limited. Available data suggest that modern INCS formulations with low systemic bioavailability are generally associated with minimal systemic exposure, which may be particularly relevant in patients with comorbidities traditionally associated with corticosteroid-related AEs. Studies evaluating INCS use during pregnancy have not demonstrated an increased risk of major congenital malformations for budesonide, FP, FF, beclomethasone, or MF when administered at recommended therapeutic doses, although available evidence remains limited. Budesonide has the most extensive pregnancy safety data among currently available INCS formulations [99].

During lactation, systemic exposure following intranasal administration is generally low, suggesting a low likelihood of clinically significant infant exposure. Nevertheless, treatment decisions during pregnancy and breastfeeding should be individualized according to symptom severity, maternal benefit, and available safety data [100].

Patients with osteoporosis, glaucoma, cataracts, or other conditions potentially affected by corticosteroid exposure may benefit from formulations characterized by very low systemic bioavailability. Current evidence reviewed in this manuscript has not demonstrated clinically meaningful effects of recommended-dose INCS therapy on bone metabolism, intraocular pressure, or cataract formation in most patients; however, clinicians should consider individual risk factors when selecting therapy and monitoring long-term treatment. In patients with significant hepatic impairment or those receiving potent CYP3A4 inhibitors, awareness of the potential for increased systemic corticosteroid exposure remains warranted. Additional studies specifically evaluating long-term safety in special populations are needed [83,84,86,96,97,98,101,102,103].

## 6. Pediatric Safety

### 6.1. Historical Concerns Regarding Growth Suppression

Long-term pediatric safety remains an important consideration in the use of INCS because of historical concerns regarding corticosteroid-associated growth suppression. Earlier studies involving BDP formulations raised concerns regarding potential reductions in growth velocity during prolonged pediatric use [89,90,91,93,94]. However, interpretation of these findings is complicated by relatively short follow-up duration, heterogeneous growth assessment methodology, and substantially greater systemic bioavailability of earlier corticosteroid formulations compared with currently used agents. Therefore, the clinical relevance of these findings remains uncertain, particularly given the lack of consistent evidence demonstrating reduced final adult height [89,90,91]. Moreover, uncontrolled AR itself may adversely affect sleep quality, daytime functioning, appetite, and overall well-being, potentially influencing growth indirectly and complicating interpretation of corticosteroid-associated growth outcomes [1].

### 6.2. Evidence for Modern Low-Systemic-Exposure INCS

In contrast, studies of currently available low-systemic-exposure INCS generally have not demonstrated clinically meaningful effects on growth or adrenal function. Budesonide studies in children with AR and asthma demonstrated growth trajectories comparable to expected reference patterns during long-term treatment [40,74,104,105]. Similarly, TAA studies in children aged 6–12 years did not identify impairment of adrenocortical function [41]. MF nasal spray studies demonstrated no significant impact on growth velocity during one year of treatment, with AE rates generally comparable to placebo [60,61,82,106,107]. Ciclesonide studies additionally demonstrated no clinically meaningful effects on growth velocity, femoral growth plates, or adrenal function in children aged 4–12 years [91,108,109]. Collectively, currently available pediatric evidence suggests that modern low-systemic-exposure formulations such as MF, FF, FP, and ciclesonide exhibit reassuring systemic safety profiles when administered at recommended therapeutic doses, although long-term real-world pharmacovigilance data remain comparatively limited.

### 6.3. Pediatric AE Profiles

Pediatric FP nasal spray studies reported predominantly mild AEs, most commonly headache, epistaxis, and upper respiratory tract infection, without evidence of clinically significant effects on growth or cortisol levels [31,110]. FF nasal spray pediatric studies identified headache, epistaxis, nasopharyngitis, pyrexia, and pharyngolaryngeal pain as the most frequently reported AEs, generally occurring at rates comparable to placebo [84,85,86,111]. Collectively, pediatric studies of modern INCS support a generally favorable safety and tolerability profile when administered at recommended therapeutic doses.

## 7. Comparative Safety Profiles of INCS

### 7.1. Older Versus Newer Formulations

Comparative studies of currently available INCS generally demonstrate broadly comparable clinical efficacy, although formulations differ in pharmacokinetic characteristics, local tolerability, and extent of systemic exposure [9,20,46]. Older agents such as BDP, budesonide, and TAA possess more extensive historical safety data and were associated with earlier concerns regarding systemic corticosteroid exposure, particularly during prolonged pediatric use [40,41,89,90,91,92,93,94]. Nevertheless, clinically significant systemic AEs remain uncommon with recommended therapeutic intranasal dosing. Studies of MF, FF, FP, and ciclesonide consistently demonstrate pharmacokinetic profiles associated with very low systemic bioavailability and limited systemic exposure across both adult and pediatric populations [15,16,19,24,25,39,59,68,82,83,84]. Systematic review evidence additionally supports the favorable efficacy and tolerability profile of ciclesonide in perennial AR [112]. Among currently available formulations, MF, FF, FP, and ciclesonide possess the strongest pharmacokinetic rationale for minimizing systemic corticosteroid exposure because of extensive first-pass metabolism and negligible oral bioavailability [15,16,19,32,47]. However, currently available comparative clinical evidence has not consistently demonstrated major differences in clinically significant systemic adverse outcomes between approved INCS formulations administered at recommended doses.

### 7.2. Local Tolerability and Sensory Characteristics

Differences in local tolerability may influence patient preference and treatment adherence. FP nasal spray formulations were associated with greater odor perception and postnasal runoff in some studies [11,12,47,75], whereas hypotonic ciclesonide formulations may reduce postnasal drip but occasionally produce greater nasal irritation [12,47,48]. Such formulation-dependent sensory characteristics may contribute meaningfully to real-world adherence despite generally comparable anti-inflammatory efficacy.

### 7.3. Clinical Relevance of Pharmacokinetic Differences

Among currently available INCS formulations, MF and FF exhibit particularly favorable pharmacokinetic characteristics because of extensive first-pass metabolism and minimal systemic absorption [16,19,24,32]. However, direct comparative evidence demonstrating clinically meaningful superiority in systemic safety outcomes between currently available formulations remains limited. Consequently, pharmacokinetic superiority should not automatically be interpreted as proven clinical superiority with respect to systemic safety outcomes [90]. Although newer low-bioavailability formulations theoretically reduce systemic corticosteroid exposure, currently available evidence generally indicates low rates of clinically significant systemic toxicity across approved INCS formulations when administered appropriately. Current evidence additionally suggests that many clinically relevant AEs associated with INCS may represent class-related phenomena influenced more by administration technique, duration of exposure, mucosal susceptibility, and formulation tolerability rather than by molecule-specific pharmacokinetic differences alone [20,73]. Overall, clinically meaningful differences between currently available INCS formulations appear to relate more to local tolerability, sensory characteristics, patient preference, and long-term adherence than to major systemic safety outcomes (Table 1). The relationship between tolerability and treatment adherence has important clinical implications extending beyond patient satisfaction alone. Sensory discomfort, local irritation, unpleasant taste, and concerns regarding AEs may contribute to treatment discontinuation or poor adherence, particularly during long-term therapy [7,8,9,10,11,12,13]. Uncontrolled AR has been associated with impaired sleep quality, reduced cognitive performance, diminished work and school productivity, and decreased overall quality of life [1]. Furthermore, AR and asthma frequently coexist as manifestations of united airway disease, and inadequate control of upper airway inflammation may adversely affect asthma control in susceptible individuals [1,113]. Consequently, selection of an INCS formulation that is both effective and well tolerated may improve long-term adherence, support sustained symptom control, and contribute to optimal overall disease management [7,52]. At a broader therapeutic-class level, recent meta-epidemiological evidence also suggests that INCS demonstrate the most favorable overall safety profile among intranasal therapies for AR [14].

### 7.4. Comparison with Previous Reviews

Our conclusions are broadly consistent with previous reviews that have reported favorable safety profiles for modern intranasal corticosteroids and low rates of clinically significant systemic adverse events [20,42]. However, the present review extends beyond traditional safety assessments by integrating pharmacokinetic determinants of systemic exposure, formulation-dependent tolerability characteristics, adherence-related considerations, pediatric safety data, and recently published evidence within a single comprehensive framework. This broader perspective highlights that although substantial pharmacokinetic differences exist among currently available formulations, these differences do not consistently translate into clinically meaningful differences in systemic safety outcomes at recommended therapeutic doses. Instead, factors such as local tolerability, sensory characteristics, administration technique, and long-term adherence may have greater practical relevance in routine clinical decision-making.

## 8. Limitations of Current Evidence

Several limitations should be considered when interpreting the current evidence regarding INCS safety. Most RCTs were relatively short in duration and may therefore have been underpowered to detect rare or delayed AEs. Definitions and reporting methodologies for AEs such as epistaxis, nasal irritation, or sensory discomfort varied substantially across studies, limiting direct comparability [14]. Many studies were industry-sponsored, potentially increasing the risk of reporting bias. In addition, direct comparative head-to-head studies between currently available low-systemic-exposure formulations remain relatively limited [90]. In addition, numerous studies relied primarily on spontaneously reported AEs rather than standardized prospective safety assessments, potentially limiting sensitivity for detection of subtle or delayed treatment-related effects. Direct head-to-head comparative safety studies between modern INCS formulations also remain limited, particularly in pediatric populations and during long-term treatment. Furthermore, many studies excluded patients with substantial comorbidity, concomitant corticosteroid exposure, or chronic polypharmacy, potentially limiting generalizability to real-world clinical practice. Furthermore, the majority of available safety data originate from studies conducted in North American and European populations, whereas evidence from Asian, African, Middle Eastern, and Latin American populations remains comparatively limited. Additional well-designed studies involving these underrepresented populations are needed to improve the generalizability of current safety data. Potential ethnic and regional differences in nasal mucosal characteristics, treatment perception, adherence patterns, and pharmacogenetic factors may influence tolerability and safety outcomes, thereby limiting the global generalizability of currently available evidence.

## 9. Future Directions

Future research should prioritize long-term real-world pharmacovigilance studies, particularly in pediatric populations and in patients requiring prolonged continuous therapy. Standardization of AE reporting, additional comparative formulation studies, and further investigation of sensory characteristics influencing adherence may improve individualized INCS selection. Further evaluation of rare systemic AEs, cumulative corticosteroid exposure, and clinically relevant pharmacokinetic interactions also remains warranted. Greater emphasis on real-world adherence, patient-reported tolerability, and administration technique may further improve individualized formulation selection and long-term treatment outcomes [7,52]. Future comparative studies should additionally evaluate patient-reported tolerability outcomes, spray technique education, real-world adherence patterns, and cumulative corticosteroid burden across multiple therapeutic routes [7,8,14,52]. Future research should also prioritize geographically and ethnically diverse study populations to better characterize potential regional differences in pharmacokinetics, tolerability, adherence patterns, and safety outcomes associated with intranasal corticosteroid therapy. Such data would improve the external validity and global applicability of current safety recommendations.

## 10. Conclusions

Currently available intranasal corticosteroids demonstrate favorable safety profiles characterized predominantly by mild local AEs and minimal clinically significant systemic toxicity. Advances in formulation design and pharmacokinetic optimization have substantially reduced systemic corticosteroid exposure, particularly with newer low-systemic-exposure agents such as MF, FF, FP, and ciclesonide. This reduction in systemic exposure is also relevant from a metabolic perspective, as systemic glucocorticoids are known to influence lipid metabolism, adipocyte biology, and metabolic homeostasis, effects that are unlikely to occur with currently available intranasal formulations administered at recommended doses. Although epistaxis and local irritation remain the most frequently reported treatment-related AEs, serious AEs and clinically meaningful endocrine complications appear uncommon across both adult and pediatric populations. Differences in formulation-dependent sensory tolerability may nevertheless influence adherence and patient preference, emphasizing the importance of individualized treatment selection during long-term management of AR. Despite substantial pharmacokinetic differences between formulations, currently available evidence does not consistently demonstrate major differences in clinically significant systemic safety outcomes at recommended therapeutic doses. Consequently, formulation selection in routine clinical practice should consider patient age, sensory tolerability, anticipated adherence, concomitant corticosteroid exposure, and patient preference, rather than relying solely on theoretical pharmacokinetic advantages.

## Figures and Tables

**Table 1:** Comparative pharmacologic, pharmacokinetic, and safety characteristics of currently available intranasal corticosteroids used in allergic rhinitis.

Characteristic	Beclomethasone Dipropionate (BDP)	Budesonide	Triamcinolone Acetonide (TAA)	Fluticasone Propionate (FP)	Fluticasone Furoate (FF)	Mometasone Furoate (MF)	Ciclesonide
Relative Systemic Bioavailability	Moderate	Moderate	Moderate	Very low	Very low	Extremely low	Extremely low
First-Pass Metabolism	Moderate	Extensive	Moderate–extensive	Extensive CYP3A4-mediated	Extensive	Extensive	Extensive CYP3A4-mediated
Glucocorticoid Receptor Affinity	Moderate	Moderate	Moderate	High	Very high	Very high	High after conversion to des-CIC
Typical dosing frequency	Twice daily	Once or twice daily	Once daily	Once or twice daily	Once daily	Once daily	Once daily
Most Common Local Adverse events	Epistaxis, nasal irritation, dryness	Nasal irritation, dryness, epistaxis	Epistaxis, nasal discomfort, irritation	Epistaxis, postnasal drip, odor perception, throat irritation	Epistaxis, nasopharyngitis, throat irritation	Epistaxis, nasal irritation, dryness	Nasal irritation, epistaxis, sensory discomfort
HPA Axis Findings	Historical concerns	No clinically meaningful suppression	No clinically meaningful suppression	No clinically meaningful suppression	No clinically meaningful suppression	No clinically meaningful suppression	No clinically meaningful suppression
Pediatric Growth Data	Historical concerns	Generally favorable	Generally favorable	Generally favorable	Generally favorable	Generally favorable	Generally favorable
Sensory tolerability	Conventional sensory profile	Generally well tolerated	Generally well tolerated	Odor perception and postnasal runoff	Favorable sensory profile	Favorable sensory tolerability	Reduced runoff with variable irritation profiles
Key Clinical Considerations	Less favorable pharmacokinetic profile	Established long-term clinical use with acceptable safety profile	Established long-term clinical use with acceptable safety profile	Low systemic bioavailability with extensive CYP3A4-mediated metabolism	Very low systemic exposure	Very low systemic exposure	Prodrug activation with minimal systemic exposure

## Data Availability

No new data were created or analyzed in this study.

## Abbreviations

The following abbreviations are used in this manuscript:

AE	Adverse event
AR	Allergic rhinitis
BDP	Beclomethasone dipropionate
CYP3A4	Cytochrome P450 3A4
Des-CIC	Desisobutyryl-ciclesonide
FF	Fluticasone furoate
FP	Fluticasone propionate
HPA axis	Hypothalamic–pituitary–adrenal axis
INCS	Intranasal corticosteroids
MF	Mometasone furoate
RCT	Randomized controlled trial
RQLQ	Rhinoconjunctivitis Quality of Life Questionnaire
TAA	Triamcinolone acetonide
TNSS	Total Nasal Symptom Score
TOSS	Total Ocular Symptom Score
